# Rapid Assessment of Cholera-related Deaths, Artibonite Department, Haiti, 2010

**DOI:** 10.3201/eid1711.110747

**Published:** 2011-11

**Authors:** Janell A. Routh, Anagha Loharikar, Marie-Délivrance Bernadette Fouché, Emily J. Cartwright, Sharon L. Roy, Elizabeth Ailes, W. Roodly Archer, Jordan W. Tappero, Thierry H. Roels, Georges Dahourou, Robert E. Quick

**Affiliations:** Centers for Disease Control and Prevention, Atlanta, Georgia, USA (J.A. Routh, A. Loharikar, E.J. Cartwright, S.L. Roy, W.R. Archer, J.W. Tappero, T.H. Roels, R.E. Quick); Laboratoire National de Santé Publique, Port-au-Prince, Haiti (M.-D.B. Fouché); IHRC, Inc., Atlanta (E. Ailes); Centers for Disease Control and Prevention, Port-au-Prince (G. Dahourou)

**Keywords:** cholera, diarrhea, mortality, bacteria, oral rehydration therapy, risk assessment, Haiti

## Abstract

We evaluated a high (6%) cholera case-fatality rate in Haiti. Of 39 community decedents, only 23% consumed oral rehydration salts at home, and 59% did not seek care, whereas 54% of 48 health facility decedents died after overnight admission. Early in the cholera epidemic, care was inadequate or nonexistent.

Epidemic cholera remains a public health problem in developing countries. In 2009, a total of 45 countries reported 221,226 cases and 4,946 deaths; for both, >98% occurred in sub-Saharan Africa (*1*).

On October 21, 2010, an outbreak of acute watery diarrhea in Artibonite and Centre Departments, Haiti, a country with no history of epidemic cholera, was confirmed as cholera when fecal specimens yielded toxigenic *Vibrio cholerae* O1 (*2*). Within 1 month, cholera spread to all 10 departments (*2*).

With prompt treatment, the cholera case-fatality rate (CFR) should remain <1% (*3*). However, by November 13, 2010, the Ministère de la Santé Publique et de la Population (MSPP) had reported 16,111 persons hospitalized with suspected cholera and 992 cholera-related deaths, for a CFR of 6.2% (*4*) (Figure 1). To determine reasons for the high CFR, we conducted a rapid cholera mortality assessment in Artibonite Department during November 12–16.

**Figure 1:**
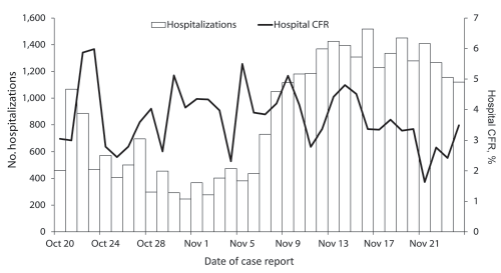
Number of and case-fatality rate (CFR) for persons hospitalized with cholera, Artibonite Department, Haiti, October 20–November 24, 2010.

## The Study

We defined cholera decedents as persons who died of suspected cholera (acute watery diarrhea in persons >5 years of age [*5*]) with illness onset after October 16, 2010, three days before the first case-patients were seen at the hospital (reflecting the 3-day average incubation period [*6*]). To locate decedents, we obtained reports of cholera-related deaths from 2 sources: admission records from 2 hospitals in Artibonite that had cholera treatment centers (Hôpital Albert Schweitzer and Hôpital Charles Colimon) and verbal reports from community health workers (CHWs). We attempted to locate households of all decedents from hospital records and verbal reports. Logistic and time constraints limited case finding to communities within 2 hours’ travel from the hospitals. We visited decedents’ households; obtained informed consent; and interviewed families about demographics, symptoms, health-seeking behavior, treatment, type of health facility, and knowledge about cholera. We also asked decedents’ household members and local CHWs about other cholera-related deaths. If additional decedents were identified, we visited their homes and interviewed household members. The Centers for Disease Control and Prevention Institutional Review Board (Atlanta, GA, USA) and MSPP determined that this emergency response activity was nonresearch.

**Figure 2:**
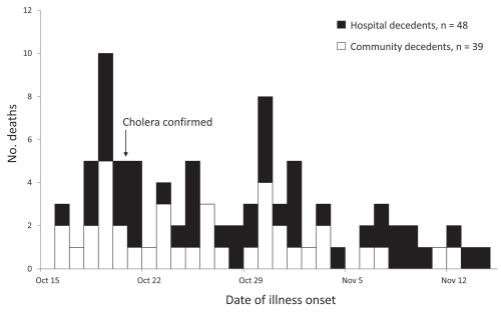
Number of persons who died of cholera, Artibonite Department, Haiti, October 16–November 14, 2010.

We enrolled 87 decedents. Of 28 decedents identified from hospital records, we found homes of 22 (79%); homes of 6 decedents could not be located or were too remote for inclusion. Illness onset ranged from October 16 through November 14; a total of 29 (33%) persons died during the first week of the epidemic (Figure 2). Median age of decedents was 50 years (range 5–100 years); 58 (67%) were male. Forty-eight (55%) decedents died in a health facility (health facility decedents) and 39 (45%) died at home or en route to a facility (community decedents). We identified 17 (35%) health facility decedents from hospital records and 31 (65%) from community interviews; we identified 5 (13%) community decedents from hospital records and 34 (87%) from community interviews.

Twenty-three (48%) health facility decedents and 9 (23%) community decedents had used oral rehydration solution (ORS) at home before seeking care (Table 1). ORS use at home was lower for persons who died during the first week of the outbreak (7 [27%]) than during the second (8 [40%]) or third (17 [46%]) weeks. We observed ORS sachets in homes of 17 (35%) health facility decedents and 14 (36%) community decedents. No respondents reported use of homemade sugar–salt solution by decedents.

**Table 1:** Reported cholera treatment received at home for health facility versus community decedents, Haiti, October–November 2010*

ORS use	Health facility decedent, no. (%), n = 48	Community decedent, no. (%), n = 39	Total, no. (%), n = 87
ORS used at home	23 (48)	9 (23)	32 (37)
Place from which ORS was obtained			
Health center	15 (65)	5 (56)	20 (63)
Pharmacy	0	1 (11)	1 (3)
Red Cross/nongovernment organization	5 (22)	0	5 (16)
Friend	3 (13)	3 (13)	6 (19)
ORS sachets observed	16 (35)	13 (35)	29 (33)

*Health facility decedent, cholera case-patient who died in a health facility; community decedent, cholera case-patient who died at home or en route to a health facility; ORS, oral rehydration solution.

Median time from illness onset to death was 20 hours (range 3 hours–7 days) for health facility decedents and 12 hours (range 2 hours–8 days) for community decedents. Twenty-two (46%) health facility decedents died on day of admission and 26 (54%) died after spending >1 night in the facility (Table 2). Twenty-three (59%) community decedents never sought care, 8 (21%) died en route to care, and 8 (21%) died after discharge. Of those who sought care, 29 (60%) health facility decedents and 7 (44%) community decedents waited <2 hours to visit a health facility. Family members of community decedents reported the following reasons for not seeking care: no need for care (19 [69%]), long distance to the health facility (6 [26%]), too ill to travel (4 [17%]), lack of transport (3 [13%]), unsafe to travel at night (3 [13%]), and cost of transport (1 [4%]).

**Table 2:** Time and location of death from cholera for health facility versus community decedents, Haiti, October–November, 2010

Time and location	Health facility decedent, no. (%), n = 48	Community decedent, no. (%), n = 39
At home before receiving care	NA	23 (59)
En route to a health facility	NA	8 (20)
On day of admission	22 (46)	NA
After overnight admission	26 (54)	NA
At home after discharge	NA	8 (20)

*Health facility decedent, cholera case-patient who died in a health facility; community decedent, cholera case-patient who died at home or en route to a health facility; NA, not applicable.

Of 48 health facility decedents, 38 (79%) were treated in hospital and 10 (21%) at a health center or dispensary. Decedents received intravenous fluids (35 [73%]), ORS (27 [56%]), both (20 [42%]), or neither (3 [9%]).

Household members of 33 (69%) health facility decedents and 30 (81%) community decedents reported receiving information about cholera after the outbreak started. The most common information sources for families of health facility and community decedents, respectively, were radio (26 [79%] vs. 26 [89%]), friend (6 [18%] vs. 8 [27%]), cellular telephone text message from MSPP (4 [12%] vs. 4 [13%]), community meeting (2 [6%] vs. 2 [7%]), and CHWs (1 [3%] vs. 3 [10%]). Fewer than half of family members of health facility (23 [48%]) and community (19 [49%]) decedents believed cholera was treatable. Of these, 16 (70%) health facility decedents and 17 (90%) community decedents knew to seek care at a health facility.

## Conclusions

Our findings suggest that, early in the cholera epidemic in Haiti, death occurred rapidly, and care was either inadequate or nonexistent. We found several possible explanations for this situation.

First, early in the outbreak, the population knew little about cholera. Many decedents did not know to seek care immediately. Knowledge, availability, and use of ORS were inadequate. Although many families acknowledged receiving cholera messages, their understanding was incomplete. Few reported receiving cholera messaging or ORS from CHWs. Global deficiencies in the distribution and use of ORS in recent years have impeded the ability of CHWs to initiate treatment (*7*).

Second, CHWs probably lacked sufficient information, experience, and resources to provide proper treatment early in the outbreak. Identification and aggressive treatment of dehydration is critical for effective cholera treatment. Deaths in health facilities in Haiti might have resulted from problems commonly observed elsewhere: overwhelming patient load, inadequate supplies, and health worker shortages (*8*).

Third, decedents’ relatives identified several commonly observed barriers to care: distance to health facility, lack of transport, and unaffordable transport (*9*). Research suggests that the effect of distance and lack of transport on cholera-related death can be mitigated by local treatment with ORS by CHWs (*10**,**11*). Finally, the epidemic strain, which was particularly virulent, might have contributed to deaths (*12*).

Active case finding detected 87% of community decedents. This finding suggests that cholera-related deaths might have been underreported, particularly in more remote communities.

Our study had several limitations. First, time and logistics limited our ability to visit remote communities where more deaths might have occurred. Second, our geographically circumscribed convenience sample might not have been representative of all cholera deaths. Third, medical records at cholera treatment facilities were incomplete or absent. Finally, our data were limited to reports from decedents’ families.

Findings from this assessment suggested several practical actions that could mitigate the risk for death from cholera. CHWs, particularly in remote settings, should receive training in cholera treatment and referral and adequate supplies of ORS; similar efforts for HIV and tuberculosis in Haiti have been promising (*13**,**14*). Health providers should receive sufficient cholera training and treatment supplies. Cholera education should be disseminated through multiple communication channels. Longer term efforts to increase health facility staffing and improve access to care should be prioritized.

In response to the epidemic, training and supplies have been provided to health workers in all 10 departments of Haiti. By April 2011, the cholera CFR had declined to <1% (www.mspp.gouv.ht/site/index.php?option=com_content&view=article&id=57&Itemid=1).
